# Efficacy and Toxicity of Bevacizumab in Children with NF2-Related Schwannomatosis: A Systematic Review

**DOI:** 10.3390/cancers17030519

**Published:** 2025-02-04

**Authors:** Annemijn L. Tops, Josefine E. Schopman, Radboud W. Koot, Hans Gelderblom, Nabila A. Putri, Latifah N. A. Rahmi, Jeroen C. Jansen, Erik F. Hensen

**Affiliations:** 1 Department of Otorhinolaryngology—Head and Neck Surgery, Leiden University Medical Center, 2333 ZA Leiden, The Netherlandse.f.hensen@lumc.nl (E.F.H.); 2 Department of Medical Oncology, Leiden University Medical Center, 2333 ZA Leiden, The Netherlands; j.e.schopman@lumc.nl (J.E.S.); a.j.gelderblom@lumc.nl (H.G.); 3 Department of Neurosurgery, Leiden University Medical Center, 2333 ZA Leiden, The Netherlands; 4 School of Medicine and Health Sciences, Atma Jaya Catholic University of Indonesia, Jakarta 14440, Indonesia; 5 Faculty of Medicine, Universitas Indonesia, Jakarta 10430, Indonesia

**Keywords:** neurofibromatosis type 2, schwannomatosis, bevacizumab, vestibular schwannoma, children, tumor response, adverse events, hearing

## Abstract

Background/Objectives: NF2-related schwannomatosis (NF2) is a tumor predisposition syndrome that typically presents with bilateral vestibular schwannomas, together with other intracranial and spinal schwannomas, meningiomas, and/or ependymomas. Bevacizumab, a VEGF inhibitor, has the potential to decrease schwannoma volume and improve hearing in adults, but the literature on the effects in children is sparse. This narrative review aims to evaluate the use of bevacizumab in pediatric NF2 patients, focusing on hearing, tumor progression, and toxicity. Methods: A literature review was conducted following PRISMA guidelines. Articles were searched in PubMed, Embase, Web of Science, Cochrane Library, Emcare, and Academic Search Premier on 18 July 2024. Inclusion criteria were patients ≤ 18 years, diagnosed with NF2 and treated with bevacizumab. Two authors independently assessed the quality of the evidence and extracted relevant data from the included articles. Results: Seventeen articles including 62 pediatric NF2 patients met the inclusion criteria. Studies varied widely in treatment regimens and outcome parameters. Tumor regression was reported in 6/56 patients (11%) and 38/56 (68%) remained stable. Hearing improved in 15/45 patients (33%) and did not further deteriorate in 27/45 (60%). An improvement in other symptoms was seen in 6/29 patients (28%). Toxicity was reported in five studies, documenting 13 adverse events in 28 patients ranging from grade 1 to grade 3. Treatment was discontinued in both patients who experienced grade 3 toxicity. Conclusions: Bevacizumab seems to be a viable treatment option for pediatric NF2 patients. Tumor regression or stabilization is achieved in the majority of patients (77%). Moreover, a considerable number of pediatric patients experience hearing stabilization or improvement (93%). Bevacizumab appears to be relatively well tolerated, offering a non-invasive therapeutic option for children with NF2 suffering from progressive vestibular schwannomas and hearing loss.

## Simple Summary

This systematic review investigates the effect of bevacizumab in pediatric patients with NF2-related schwannomatosis (NF2), a tumor predisposition syndrome characterized by the occurrence of multiple schwannomas, meningiomas, and/or ependymomas, with bilateral vestibular schwannomas as the hallmark lesion. While bevacizumab has demonstrated effectiveness in reducing the size of (vestibular) schwannomas and improving hearing in adults, its efficacy in children remains underexplored. Through a comprehensive review of the existing literature, we found that the majority of the reported pediatric patients experienced regression or stabilization of the target tumor. A considerable number of pediatric patients experienced hearing stabilization or even improvement. Bevacizumab was relatively well tolerated, with only a few reported severe adverse effects. These findings suggest that bevacizumab offers a promising, non-invasive therapeutic option for progressive (vestibular) schwannomas and/or hearing loss in children with NF2.

## 1. Introduction

NF2-related schwannomatosis (NF2, formerly neurofibromatosis type 2) is a rare, autosomal dominant tumor predisposition syndrome caused by a mutation in the NF2 gene. The incidence is estimated to be between 1 in 25,000 to 33,000 in the United Kingdom and 1 in 87,410 in Finland [1,2]. NF2 typically presents with bilateral vestibular schwannomas but is associated with numerous other tumors, including meningiomas, ependymomas, and intracranial, intraspinal, and peripheral schwannomas [3]. The clinical phenotype varies widely, depending on the type of genetic mutation [4]. Symptoms can manifest at any age, but the peak incidence occurs during adolescence and early adulthood, while occurrences in young children are less common [5]. However, when NF2 manifests early in life, it tends to be more severe, with a higher likelihood of developing multiple and progressive tumors [6]. Consequently, optimal management of tumors in pediatric patients is of high importance. In adults, current management options comprise active surveillance, surgery, stereotactic radiotherapy, and pharmacotherapy, and the treatment strategy is tailored to individual patient and tumor characteristics [7,8,9]. In pediatric patients, however, treatment approaches may differ due to potential long-term adverse events, which are of greater concern in this population [10]. Additionally, because pediatric patients often present with a greater tumor burden, a systemic treatment that targets multiple tumors simultaneously—rather than addressing them individually through surgery or radiotherapy—may be more beneficial.

Bevacizumab, a VEGF inhibitor, has shown promise in reducing both hearing loss and tumor volume, primarily in adult populations [8]. However, evidence regarding its effects on hearing, tumor progression, and toxicity in children is limited. This study aims to provide a comprehensive review of the currently available evidence regarding the use of bevacizumab in pediatric patients with NF2-related schwannomatosis, with a focus on its effects on hearing, tumor progression, and treatment-related toxicity.

## 2. Materials and Methods

### 2.1. Search Strategy

Following the PRISMA guidelines, an extensive literature review was performed to analyze the effect and toxicity of bevacizumab in children. Relevant articles were identified in PubMed, Embase, Web of Science, Cochrane Library, Emcare, and Academic Search Premier through a computerized search conducted by an academic librarian. The search strategy included the MeSH terms ‘Neurofibromatosis 2’ and ‘Bevacizumab’ along with their corresponding entry terms. The limited amount of available literature allowed the search strategy to remain broad, without narrowing the search with terms such as ‘child’ or ‘pediatric’. This approach ensured that studies comprising both adults and children were included in this search, and pediatric subgroups within these studies were not overlooked and could be included in this review when separate extraction of data for this population was possible. The complete search strategy is listed in Supplementary Table S1. The search was conducted on 18 July 2024, without restrictions on language or date of publication. After removing duplicates, the remaining abstracts were screened independently by two reviewers, and discrepancies were resolved by a third reviewer. Full texts were then assessed for eligibility. This systematic review was not registered and a protocol was not published.

### 2.2. Inclusion and Exclusion Criteria

Inclusion criteria were (1) patients aged ≤18 years, (2) diagnosed with NF2, (3) treated with bevacizumab, and (4) reported radiological, clinical, and/or hearing outcomes and/or toxicity. Exclusion criteria were (1) studies describing administration of bevacizumab via routes other than intravenous infusion and (2) studies that did not report outcomes specifically for children. Studies that included mixed populations were included if data of the pediatric subgroup could be extracted separately.

### 2.3. Data Extraction

Data were extracted by two reviewers using a data extraction form. Information collected from each study included the first author, year of publication, study design, total number of patients, and number of pediatric patients. Information collected from each eligible patient included age, gender, dosage, duration of treatment, baseline tumor size, previous treatments, radiographic response, hearing response, clinical response, and complications. In the study where the data were only presented in graphs, data points were extracted manually [11].

### 2.4. Assessment of Quality and Risk of Bias

Assessment of quality of evidence and risk of bias was assessed by two reviewers independently, and discrepancies were resolved by a third reviewer. The quality of the included cohort studies and case series was assessed using a version of the Newcastle Ottawa Scale, modified for non-comparative case series; see Supplementary Table S1. The quality of included case reports was assessed using the JBI Critical Appraisal Tool for case reports; see Supplementary Table S2.

### 2.5. Data Analysis

Due to the high level of heterogeneity of the included studies—such as differences in dosage, length of treatment, outcome measures and previous treatments—a meta-analysis on pooled data could not be performed. Consequently, a narrative synthesis was conducted, with an emphasis on summarizing and comparing the findings related to efficacy and toxicity.

## 3. Results

### 3.1. Overview of Main Characteristics of Eligible Studies

**Figure 1:**
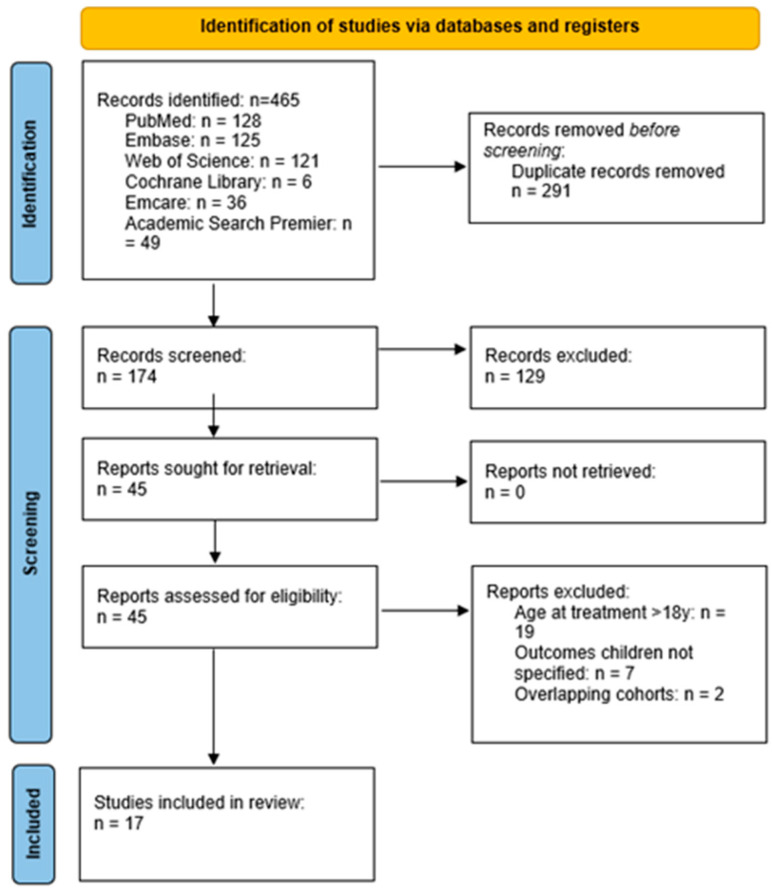
Flowchart illustrating the selection process of the studies according to the Preferred Reporting Items for Systematic Reviews and Meta-Analyses (PRISMA) guidelines.

The search strategy yielded 465 studies. After removing the duplicates, 174 abstracts were screened for eligibility. Subsequently, 129 studies were excluded based on the predefined criteria. After assessing the 45 full articles, 17 articles comprising 62 pediatric NF2 patients remained that met the inclusion criteria. Details on reasons for exclusion are stated in Figure 1.

### 3.2. Study Characteristics

The seventeen studies were published between 2009 and 2023 with baseline characteristics presented in Table 1. The total number of described patients per study varied between 1 and 61, and the number of pediatric patients fulfilling the inclusion criteria ranged between 1 and 17 per study. Dosage was variable and ranged from 5 to 15 mg/kg every 2 to 4 weeks. Duration of treatment ranged between 3 and 86 months. Baseline tumor size varied between 0.39 and 38.9 cm^3^. Previous therapy, either systemic treatment or interventions targeting vestibular schwannomas specifically, was reported in eleven studies [8,11,12,13,14,15,16,17,18,19,20].

### 3.3. Methodological Quality

The results of the critical appraisal tools used are presented in Supplementary Tables S1 and S2. Overall quality was moderate in seven studies and high in ten.

### 3.4. Radiological Response

Radiological response, as shown in Table 2, was measured in all studies including 62 patients, although the methods for reporting varied widely. Some studies reported the response only in descriptive terms (tumor progression, stable disease, or tumor regression) while other studies included numerical measurements (volume change in percentage or in cm^3^). In most studies, progressive disease was defined as >20% increase in tumor volume compared to baseline volume measurements of VS and tumor regression was defined as >20% reduction in tumor volume. One study used linear measurements to assess radiological response [19]. Three studies did not specify whether tumor size was measured using linear or volumetric measurements, nor did they provide baseline tumor measurements [13,15,18].

Radiological response was detailed per patient in all studies except one [24]. In that study, including six pediatric participants and ten corresponding VS, the response was reported per tumor. Of the ten VS, five (50%) showed tumor progression and five (50%) showed stable disease. Since no percentage of patients could be extracted from these data on the specific tumors, results are shown in Table 1 but could not be included in the calculation. In patients with two tumors with different radiological responses, the most favorable response was included [11].

In the sixteen remaining studies, 38 of 56 patients (68%) were reported to have stable disease. Regression in tumor size was reported in six (11%) patients and progressive disease was reported in five (9%). Two studies, comprising seven patients (13%), only reported ‘no response’, not specifying whether it meant tumor stability or progression of disease [22,23].

### 3.5. Hearing Response

Thirteen studies assessed hearing response, shown in Table 3. Hearing was described in various ways in these studies. Some reported changes in word recognition score (WRS) in percentage or change in pure tone average (PTA) in decibels (dB), or both. Other studies offered only descriptive outcomes, indicating whether patients experienced progressive hearing loss, stable hearing, or hearing improvement. In most studies, hearing improvement was defined as a >10 dB improvement in PTA or a significant increase in WRS compared to baseline scores. However, two studies adopted stricter definitions, requiring a >20 dB and >12 dB improvement in PTA, respectively [17,24]. Hearing was assessed according to the definition used in the study. In one study, in which hearing was assessed using both PTA and WRS, the best response was included [11]. In another study also assessing both PTA and WRS, only the response in PTA was included, since the assessment of WRS was solely provided per tumor and not per patient [24].

The thirteen studies that reported on hearing outcomes comprised 45 patients. Hearing improved in 15 patients (33%), remained stable in 27 patients (60%), and worsened in 3 patients (7%).

### 3.6. Symptomatic Response

Symptomatic response was reported in six studies involving a total of 29 patients; however, the symptoms were not specified in the majority of these. In eight patients (28%), symptoms improved, while they remained stable in twenty-one patients (72%). Of these eight patients, one reported improved dysphagia and another reported improvement in sensory deficits and pain [21]. The remaining six patients were reported to have a mild improvement in subjective hearing and reduction in tinnitus [17]. In the 21 patients with stable symptoms, the stability was observed in different domains. Four patients were specifically described to have a stable neurological examination [13,15,16]. Eleven patients were only mentioned to have a stable clinical response, with no further details provided about the specific symptoms [17]. Six patients showed no clinically significant difference in questionnaires assessing tinnitus-related complaints (TRQs) and quality of life (NFTI-QoL) [24].

### 3.7. Toxicity

Toxicity was documented specifically for children in five studies comprising 28 patients. Thirteen adverse events were reported, ranging from grade 1 to grade 3 according to the Common Terminology Criteria for Adverse Events (Table 4). Two patients experienced a grade 3 adverse event (one osteomyelitis and one hypertension) and discontinued treatment because of it [14]. No other patients discontinued treatment due to toxicity. Grade 1 adverse events included chest pain, epistaxis, malaise, and a non-infectious wound complication. The grade 2 adverse events included intermenstrual bleeding, proteinuria, epistaxis, hypertension (*n* = 2), hypothyroidism, and secondary amenorrhea. Two studies reported no significant side effects [16,25].

## 4. Discussion

This study reviews the published experience with bevacizumab treatment in children affected by NF2. While there is limited research on the efficacy of bevacizumab in adults, specific results for pediatric patients are even more scarce. This study addresses an important gap in the current literature regarding the treatment of NF2 in children, who often present with a more severe disease phenotype and limited treatment options. To date, only 62 pediatric patients receiving bevacizumab have been reported separately. There is considerable heterogeneity in the existing studies concerning the doses of bevacizumab administered, duration of treatment, and reported outcome parameters. This variability precludes a meta-analysis of the outcome of treatment on pooled data.

The therapeutic effect of bevacizumab appears to be less pronounced in children compared to adults. A 2021 meta-analysis by Shi et al. pooled data of 274 NF2 patients with 332 VS and reported tumor regression in 30% of patients [27]. A more recent systematic review by Chiranth et al., which assessed targeted therapy for VS in NF2 patients, included 10 studies with 200 patients aged 10 to 79 and found tumor regression in 38% and tumor progression in 9% of patients [28]. These results suggest that bevacizumab has a more significant effect on tumor volume in adult patients than in pediatric patients, as our review shows tumor regression in only 11% and progression in 9–22%. However, hearing outcomes appear more comparable between the age groups. Shi et al. reported a positive hearing response in 32% of patients, whereas Chiranth et al. documented hearing improvement in 45% and hearing decline in 10% of cases. Our review reveals hearing improvement in 33% of pediatric patients and a decline in 7%. This suggests that bevacizumab offers comparable hearing benefits in both children and adults. However, because of the small numbers and differences in patient characteristics, study design, and outcome parameters, comparisons between effectiveness in children and adults should be made with caution. Future studies including standardized outcome measures and larger patient cohorts will be necessary to adequately compare these patient groups. One possible explanation for the reduced effectiveness of bevacizumab on tumor progression in children may be attributed to the more progressive course of the disease in younger patients [29,30,31]. Another factor may be the definition of treatment success. This hypothesis is illustrated by the findings of Hochart et al. who described two patients with a persistent increase in tumor volume after bevacizumab, although the progression rate showed a substantial decrease [14]. The tumor progression rate in both patients decreased by more than threefold; for example, one patient’s tumor progression rate decreased from 123% to 36%. Despite the decrease in progression rates, these tumors are still progressive and therefore labeled therapy failures, whereas the decrease in the tumor progression rate could also be considered a therapy success. This discrepancy illustrates that, when drawing conclusions from the most frequently used outcome parameters, the effect of bevacizumab might be underestimated.

Toxicity is a primary concern when considering bevacizumab treatment in children. Based on the adverse events reported in the included studies, bevacizumab appears to be reasonably well tolerated. Toxicity could be evaluated in 28 children, and whilst 13 adverse events were identified, most were mild to moderate. Only two grade 3 adverse events were found, both leading to treatment discontinuation. The incidence of severe adverse (grade 3 or 4) events in children (7%; 2 out of 28) appears lower than the rates reported in the two meta-analyses comprising predominantly adult patients (12% and 17%) [27,32]. Hypertension of all severities occurred in 11% of pediatric patients (3 out of 28), which is lower than the 29% and 33% observed in adults. Additionally, proteinuria is less common in children (4%; 1 out of 28) compared to adults (30–43%). These lower rates of adverse events may in part be attributed to the reduced presence of comorbidities in children. However, comparisons between adult and pediatric patients are hampered by the limited number of pediatric patients for which toxicities are specified, thus precluding definite conclusions.

Long-term toxicity is of particular importance in children receiving bevacizumab. While more data on long-term toxicity in this group of patients is needed, four studies in this review included treatment durations exceeding two years. Notably, 11 out of 13 reported adverse events were observed in the studies with the longest treatment durations. This observation suggests a correlation between cumulative dosage and the incidence of adverse events. A study by Slusarz et al., investigating long-term toxicity in adults, found that while overall toxicity was manageable, the cumulative dose of bevacizumab was associated with increased blood pressure [33]. Although hypertension, one of the most prevalent adverse events, was observed in 58% of patients at a median treatment duration of 34 months, it was successfully managed with medication in 39% of adult patients, and no patients required interruption in therapy. Proteinuria had an incidence of 62% in adult patients but was not associated with cumulative bevacizumab dosage. Farschtschi et al. found that by decreasing the dosage in a study of three long-term bevacizumab users, toxicity could be managed and effectiveness maintained [34]. No other studies have been published to date investigating the effects and toxicity of smaller doses than 2.5 mg/kg every 3 weeks. This could be a potential solution worth exploring, especially given the increasing number of patients requiring long-term bevacizumab treatment. This possibility is further supported by the meta-analysis conducted by Shi et al., which separately analyzed low-dose and high-dose effectiveness. The analysis confirms that high-dose regimen increases the risk of adverse events in adult patients without improving radiological or hearing response. Another possible way to manage adverse events is introducing treatment breaks, allowing for resolution of treatment toxicities in between cycles. It has been shown that bevacizumab treatment can be restarted successfully after discontinuation in NF2 patients; however, this has not yet been studied in children specifically [34,35].

The limitations of this review include the limited number of retrospective cohort studies and case series and the absence of randomized controlled trials. In addition, some studies only reported on short observation periods, precluding adequate evaluation of long-term effects and toxicities. In general, there is a lack of detailed information regarding the course of the treatment response per patient over time, including specific time points at which tumor progression or stability were assessed. It is well established that tumors may respond differently with each treatment cycle, initially causing a reduction in tumor size, followed by no effect, or vice versa [35]. Therefore, data on tumor size at multiple time points and the impact of treatment breaks would provide valuable insights into the overall response. Another limitation of the study is the heterogeneity in outcome measures across studies, which hampers comparability between studies and precludes the pooling of data. To draw clear conclusions, particularly in small populations affected by rare diseases, it is essential that study design and outcomes are reported in a standardized manner. Evaluation of the therapeutic interventions in NF2 should encompass effect on tumor size, hearing, toxicity, and symptomatic response, including vestibular symptoms. The current literature contains data on substantially more children than the 62 patients in this review; however, these outcomes are often not stratified by age. As this review shows that there may be distinct differences in the effects of bevacizumab in pediatric and adult patients, we argue that when studies include both patient subgroups, results should be presented separately.

## 5. Conclusions

Bevacizumab appears to be a viable treatment option for pediatric patients with NF2. Stabilization or regression of the target tumor is achieved in the majority of patients (77%), which is especially important since children often have faster growing tumors. Moreover, following treatment with bevacizumab, hearing did not further deteriorate in a remarkable 93%, and in 33%, hearing even improved. Bevacizumab appears to be relatively well tolerated, offering a non-invasive therapeutic option for children facing multiple growing tumors and hearing loss. Future research on the effects and long-term toxicity in pediatric patients is needed and should include the use of uniform treatment regimens and standardized outcome measures.

## Figures and Tables

**Table 1:** Main characteristics of the studies included in the review.

Study	Design	Pediatric Patients (Total Patients)	Pediatric Patients with Radiological Outcome Reported	Pediatric Patients with Hearing Outcome Reported	Median Age at Treatment (Range; Years)	Gender F (%)	Initial Dosage	Median Duration of Treatment (Range; Months)	Median Baseline Tumor Size (Range)	Previous Treatment for VS
Ardern-Holmes et al., 2021 [21]	Retrospective cohort	4 (11)	4	3	14.5 (13–17)	NS	5–15 mg/kg/2–4 weeks (NS)	13.5 (9–47)	7.6 (1.45–37.9) cm^3^	NS
Farschtschi et al., 2016 [22]	Retrospective cohort	2 (8)	2	0	15	0	7.5 mg/kg/3 weeks; 5 mg/kg/2 weeks	34.5 (11–58)	NR	NR
Fuji et al., 2020 [12]	Feasibility study	3 (10)	3	0	18 (12–18)	2 (66)	5 mg/kg/2 weeks	6	5.1 (0.81–16.16) cm^3^	SRS (*n* = 1); SRT (*n* = 1)
Gugel et al., 2019 [11]	Retrospective cohort	2 (9)	2	1	17.5 (17–18)	2 (100)	5 mg/kg/2 weeks	28 (23–33)	0.4 (0.39–1.17) cm^3^	Surgery (*n* = 2)
Hawasli et al., 2013 [13]	Case series	2 (6)	2	2	15 (14–16)	0	5–10 mg/kg/2–3 weeks (NS)	8.5 (5–12)	NR	Surgery + CI (*n* = 1); lovastatin (*n* = 1)
Hochart et al., 2015 [14]	Retrospective cohort	7 (7)	7	4	15.0 (11–18)	4 (57)	5–10 mg/kg/2 weeks	11.3 (3–55)	2.7 (0.4–13.5) cm^3^	Surgery (*n* = 3)
Kim et al., 2022 [15]	Case report	1 (1)	1	0	NR	0	5 mg/kg/2 weeks	6	NR	None
Morris et al., 2016 [23]	Prospective cohort	6 (61)	6	0	NR	NS	5–10 mg/kg/2–3 weeks	NS	5.0 (1.6–8.9) cm^3^	NS
Nigro et al., 2020 [16]	Case report	1 (1)	1	1	13	0	5 mg/kg/3 weeks	108	NR	None
Plotkin et al., 2009 [8]	Retrospective cohort	3 (10)	3	3	18 (16–18)	1 (33)	5 mg/kg/2 weeks	13 (8–19)	24.9 (22.6- 38.9) cm^3^	Erlotinib (*n* = 2)
Plotkin et al., 2023 [24]	Prospective cohort	6 (20)	6	6	17 (12–18)	4 (67)	5 mg/kg/3 weeks *	17 (14.5–18)	NS	NS
Renzi et al., 2020 [17]	Retrospective cohort	17 (17)	17	17	15.8 (10–17)	9 (53)	5–10 mg/kg/2–3 weeks	14.6 (3.6–86.4)	NR	Surgery; SRS; RTx and erlotinib/sirolimus
Santoro et al., 2019 [18]	Case report	1 (1)	1	1	13	0	5 mg/kg/2 weeks	6	NR	None
Shepard et al., 2012 [19]	Case series	2 (5)	2	2	NR	1 (50)	NR	18	1.7 (1.5–3.9) cm **	Surgery (*n* = 2)
Subbiah et al., 2012 [25]	Case series	1 (5)	1	1	16	1 (100)	NR	10+	NR	NR
Sverak et al., 2019 [26]	Case series	3 (17)	3	3	17 (15–18)	2 (67)	5–10 mg/kg/2–6 weeks (NS)	9 (6–9)	0.3 (0.3–4.5 cm^3^	NR
Tripathi et al., 2020 [20]	Case series	1 (2)	1	1	17	1 (100)	NR	8 cycles	10.21 cm^3^	GKRS
Total patients		62	62	45						

NR = not reported, NS = not stated specifically for pediatric patients, VS = vestibular schwannoma, GKRS = gamma knife radiosurgery, SRS = stereotactic radiosurgery, SRT = stereotactic radiation therapy, RTx = radiotherapy. * Patients started with an induction dosage of 10q2 and after 6 months switched to maintenance dosage of 5q3; present study includes results on maintenance dosage of 5q3. ** Only linear measurements of tumor size were reported, instead of volumetric measurements.

**Table 2:** Reported radiological response after treatment with bevacizumab in pediatric patients with NF2-related schwannomatosis.

Study	Tumor Regression (Number of Patients; %)	Change in Tumor Volume (%)	Stable Disease (Number of Patients; %)	Change in Tumor Volume (L/R; %)	Tumor Progression (Number of Patients; %)	Change in Tumor Volume (L/R; %)	Stable Disease or Tumor Progression (Number of Patients; %)	Total Eligible Patients
Ardern-Holmes et al., 2021 [21]			2 (50%)	+17%; +14%	2 (50%)	+30%; +241%		4
Farschtschi et al., 2016 [22]							2 (100%)	2
Fuji et al., 2020 [12]			3 (100%)	+19/−15%; +13/+1%; +6/+2%				3
Gugel et al., 2019 [11]			2 (100%)	+0% *; +23%/+4% *				2
Hawasli et al., 2013 [13]			2 (100%)					2
Hochart et al., 2015 [14]	1 (14%)	−22%	4 (57%)	−17%; +12%; +0%; −3%/−6%	2 (29%)	+51%; +36%/+81%;		7
Kim et al., 2022 [15]			1 (100%)					1
Morris et al., 2016 [23]	1 (17%)						5 (83%)	6
Nigro et al., 2020 [16]			1 (100%)					1
Plotkin et al., 2009 [8]	1 (33%)	−44%	2 (67%)	−5%; −17%				3
Plotkin et al., 2023 [24]			5/10 tumors (50%)		5/10 tumors (50%)			0
Renzi et al., 2020 [17]	2 (12%)		15 (88%)					17
Santoro et al., 2019 [18]			1 (100%)					1
Shepard et al., 2012 [19]			2 (100%)	0%; −15%				2
Subbiah et al., 2012 [25]			1 (100%)					1
Sverak et al., 2019 [26]	1 (33%)	−20%	1 (33%)	−15%	1 (33%)	+74%		3
Tripathi et al., 2020 [20]			1 (100%)					1
Total	6 patients (11%)		38 patients (68%)		5 patients (9%)		7 patients (13%)	56 patients

* = extracted manually from graph.

**Table 3:** Reported hearing response after treatment with bevacizumab in pediatric patients with NF2-related schwannomatosis. Only studies reporting data on hearing response are included.

Study	Hearing Improvement (Number of Patients; %)	Stable Hearing (Number of Patients; %)	Hearing Deterioration (Number of Patients; %)	Total Eligible Patients
Ardern-Holmes et al., 2021 [21]		2 (67%)	1 (33%)	3
Gugel et al., 2019 [11]		1 (100%) ***		1
Hawasli et al., 2013 [13]	1 (50%)	1 (50%)		2
Hochart et al., 2015 [14]	1 (25%)	3 (75%)		4
Nigro et al., 2020 [16]		1 (100%)		1
Plotkin et al., 2009 [8]	1 (33%)	2 (67%)		3
Plotkin et al., 2023 [24]	1 (17%) **	4 (67%) **	1 (17%) **	6
Renzi et al., 2020 [17]	8 (47%) *	9 (52%) *		17
Santoro et al., 2019 [18]		1 (100%)		1
Shepard et al., 2012 [19]	1 (50%)	1 (50%)		2
Subbiah et al., 2012 [25]		1 (100%)		1
Sverak et al., 2019 [26]	1 (33%)	1 (33%)	1 (33%)	3
Tripathi et al., 2020 [20]	1 (100%)			1
Total	15 (33%)	27 (60%)	3 (7%)	45

* = Alternate definition of hearing response PTA > 20 dB; ** = alternate definition of hearing response PTA > 12 dB; *** = extracted manually from graph.

**Table 4:** Reported toxicity of bevacizumab in pediatric patients with NF2-related schwannomatosis. Only studies reporting relevant data are included.

Study	Adverse Events (Frequency)	Grade	Consequence	Total Eligible Patients
Hawasli et al., 2013 [13]	Chest pain (1)	1	None	2
	Epistaxis (1)	1	None	
Hochart et al., 2015 [14]	Osteomyelitis (1)	3	Discontinuation of treatment	7
	Intermenstrual bleeding (1)	2	None	
	Hypertension (1)	3	Discontinuation of treatment	
	Proteinuria (1)	2	None	
	Malaise (1)	1	None	
	Non-infectious wound infection (1)	1	None	
	Epistaxis (1)	2	None	
Nigro et al., 2020 [16]	None		None	1
Renzi et al., 2020 [17]	Hypertension (2)	2	None	17
	Hypothyreoidism (1)	2	None	
	Secondary amenorrhea (1)	2	None	
Subbiah et al., 2012 [25]	None		None	1
Total	13			28

## Data Availability

No new data were created or analyzed in this study. Data sharing is not applicable to this article.

## Supplementary Materials

The following supporting information can be downloaded at https://www.mdpi.com/article/10.3390/cancers17030519/s1: Table S1: Quality of evidence assessment of non-comparative cohorts and case series; Table S2: Quality of evidence assessment of case reports.
