# Supplementary material for: Efficacy and Toxicity of Bevacizumab in Children with NF2-Related Schwannomatosis: A Systematic Review
Source: Cancers (Basel). 2025 Feb 4;17(3):519. doi: 10.3390/cancers17030519 (PMC11817438; doi:10.3390/cancers17030519)
Supplement: Supplementary file 1 [file cancers-17-00519-s001.zip › cancers-3385653-supplementary.pdf]

Supplementary Table 1 – Quality of Evidence Assessment of non-comparative cohorts and case series.

Study	Criterion	1	2	3	4	5	Total	Overall Quality
**Ardern-Holmes et al. 2021**		1	1	1	1	1	5	High
**Farschtschi et al. 2016**		1	1	1	1	1	5	High
**Fuji et al. 2020**		1	1	1	1	1	5	High
**Gugel et al. 2019**		0	1	1	1	1	4	Moderate
**Hawasli et al. 2013**		0	1	1	1	1	4	Moderate
**Hochart et al. 2015**		1	1	1	1	1	5	High
**Morris et al. 2016**		1	1	1	1	1	5	High
**Plotkin et al. 2009**		1	1	1	1	1	5	High
**Plotkin et al. 2023**		1	1	1	1	1	5	High
**Renzi et al. 2020**		1	1	1	1	1	5	High
**Shepard et al. 2012**		0	1	1	0	1	3	Moderate
**Subbiah et al. 2012**		1	1	1	1	1	5	High
**Sverak et al. 2019**		1	1	1	1	1	5	High

Modified Newcastle-Ottawa Quality Assessment Scale

1. Did the patient(s) represent the whole case(s) of the medical center?
2. Was the correct diagnosis made?
3. Was follow-up long enough for outcomes to occur?
4. Were all important data cited in the report?
5. Was the outcome correctly ascertained?

Supplementary Table 2 - Quality of Evidence Assessment of case reports

Study	Criterion	1	2	3	4	5	6	7	8	Total	Overall Quality
**Kim et al. 2016**	Case report	1	0	0	1	1	1	0	1	5	Moderate
**Nigro et al. 2019**	Case report	0	0	0	1	1	1	1	1	5	Moderate
**Santoro et al., 2019**	Case report	1	1	1	0	1	1	0	1	6	Moderate
**Tripathi et al. 2020**	Case report	1	1	1	1	0	1	0	1	6	Moderate

1. Were patient's demographic characteristics clearly described?
2. Was the patient's history clearly described and presented as a timeline?
3. Was the current clinical condition of the patient on presentation clearly described?
4. Were diagnostic tests or assessment methods and the results clearly described?
5. Was the intervention(s) or treatment procedure(s) clearly described?
6. Was the post-intervention clinical condition clearly described?
7. Were adverse events (harms) or unanticipated events identified and described?
8. Does the case report provide takeaway lessons?
